# Supplementary material for: Functional compensation precedes recovery of tissue mass following acute liver injury
Source: Nat Commun. 2020 Nov 19;11:5785. doi: 10.1038/s41467-020-19558-3 (PMC7677389; doi:10.1038/s41467-020-19558-3)
Supplement: Supplementary file 1 — Supplementary Information [file 41467_2020_19558_MOESM1_ESM.pdf]

Supplementary Information

Functional Compensation Precedes Recovery of Tissue Mass Following Acute Liver Injury

Walesky, et al.

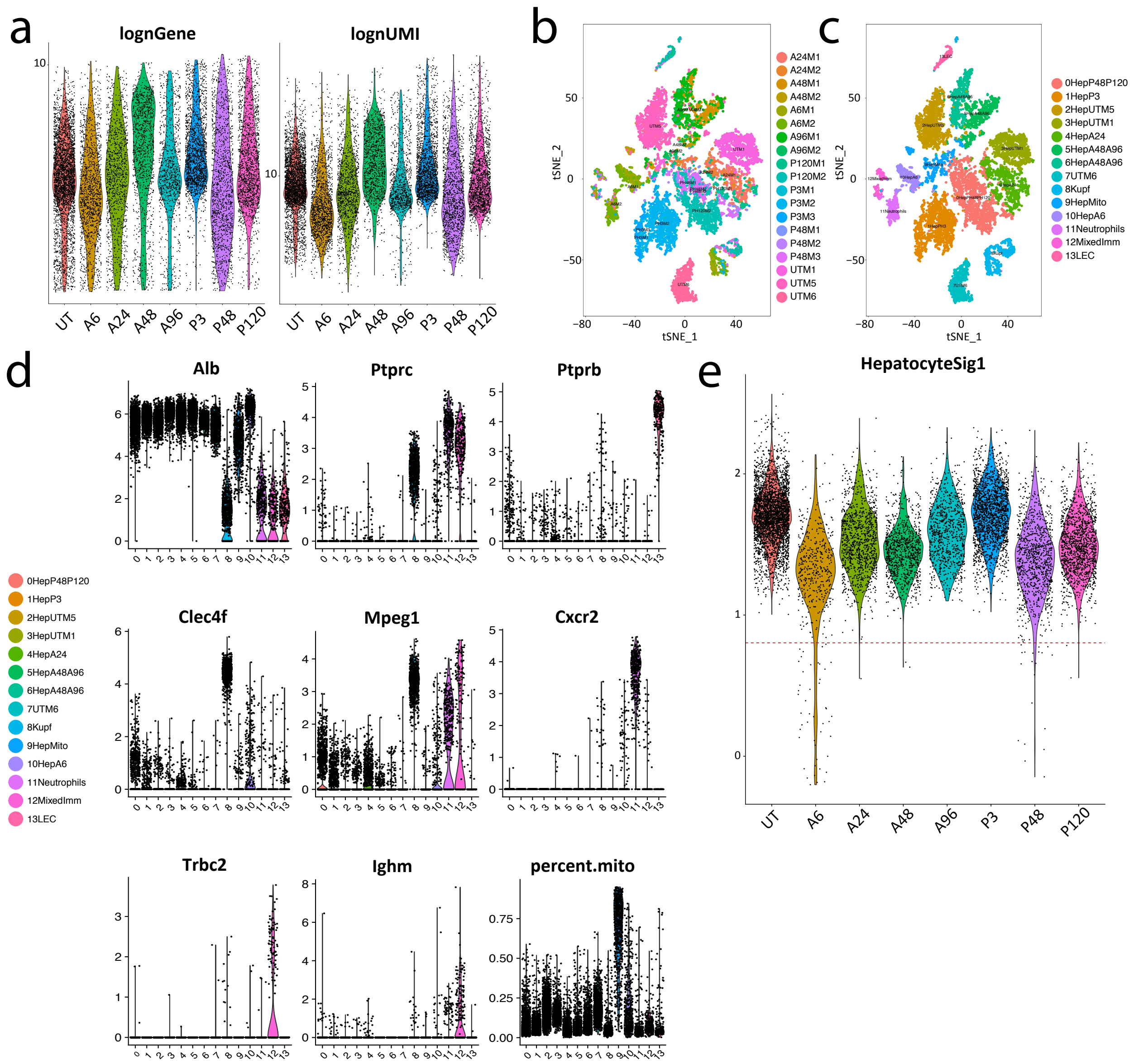

Supplementary Fig. 1 | scRNA-Seq Data Processing

(a) log(nGene) and log(nUMI) for each treatment condition. **(b)** t-SNE colored by mouse of origin. **(c)** t-SNE colored by cluster. Clusters are numbered from most to fewest member cells and annotated by cell type. **(d)** Violin plots for marker gene expression and percent mitochondrial content (percent.mito) in each cluster. Normalized expression [ln(tp10K+1)]. **(e)** Hepatocyte Signature Scores for cells in good quality hepatocyte clusters, grouped by treatment condition. Cells scoring less than 3 standard deviations below the mean (dashed red line) were filtered out as non-hepatocytes. Remaining cells were included in the high-quality hepatocyte dataset for further analysis.

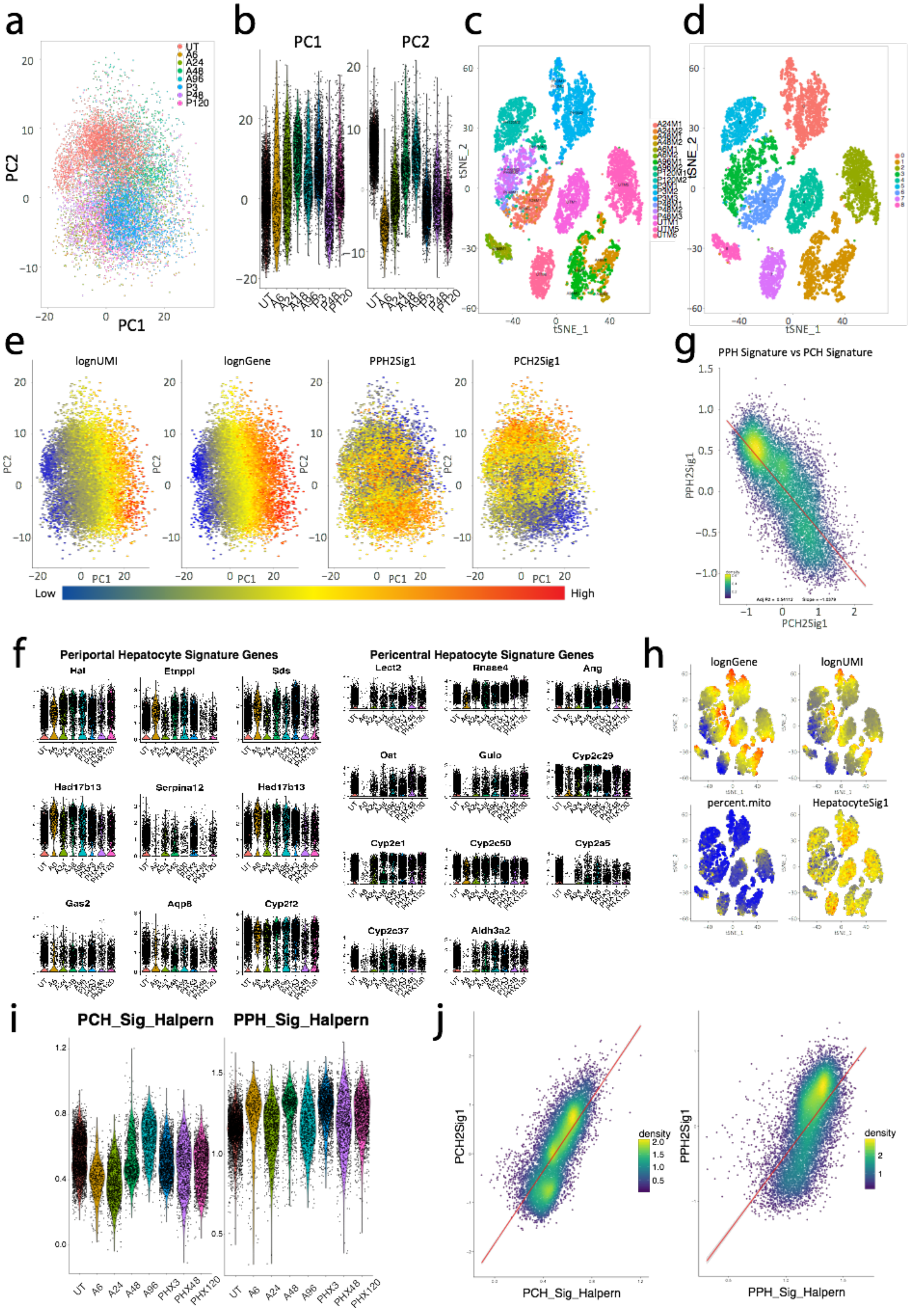

a
b
c
d
e
f
g
h
i
j
PC1
PC2
tSNE_1
tSNE_2
lognUMI
lognGene
PPH2Sig1
PCH2Sig1
Low
High
PPH Signature vs PCH Signature
Periportal Hepatocyte Signature Genes
Pericentral Hepatocyte Signature Genes
percent.mito
HepatocyteSig1
PCH_Sig_Halpern
PPH_Sig_Halpern
density

Supplementary Fig. 2 | Hepatocyte dataset analysis

(a) Principal Component Analysis (PCA) of hepatocyte dataset, PC1, PC2. Cells (dots) colored by treatment condition. **(b)** Violin plot of PC1 and PC2 scores for each cell, grouped by treatment condition. **(c)** t-sne, colored by mouse of origin. **(d)** t-SNE colored by SNN clustering assignment. **(e)** PCA (PC1, PC2), colored by lognUMI, lognGene, Periportal Hepatocyte (PPH) Signature, and Pericentral Hepatocyte (PCH) Signature. Blue, low; yellow, medium; red, high. **(f)** Violin plots of genes used to calculate PPH Sig and PCH Sig, grouped by treatment condition. Normalized expression [ln(tp10K+1)]. **(g)** Plot of PPH Signature vs PCH Signature for each cell in the dataset. R2 regression line shown in red. Color represents density of points from low (blue) to high (yellow). **(h)** t-SNE colored by lognGene, lognUMI, percent mitochondrial content (percent.mito) and Hepatocyte Signature Score. Blue, low; yellow, medium; red, high. **(i)** Violin plots of PCH_Signature_Halpern (left) and PPH_Signature_Halpern (right) for each cell, grouped by treatment condition. **(j)** Plot of correlation derived PCH Signature (left) or PPH Signature (right) vs respective Halpern-derived signature (Methods) for each cell in the dataset. R2 regression line shown in red. *(PCH: R2=0.567, p < 10e-5; PPH R2=0.399, , p < 10e-5)* Color represents density of points from low (blue) to high (yellow).

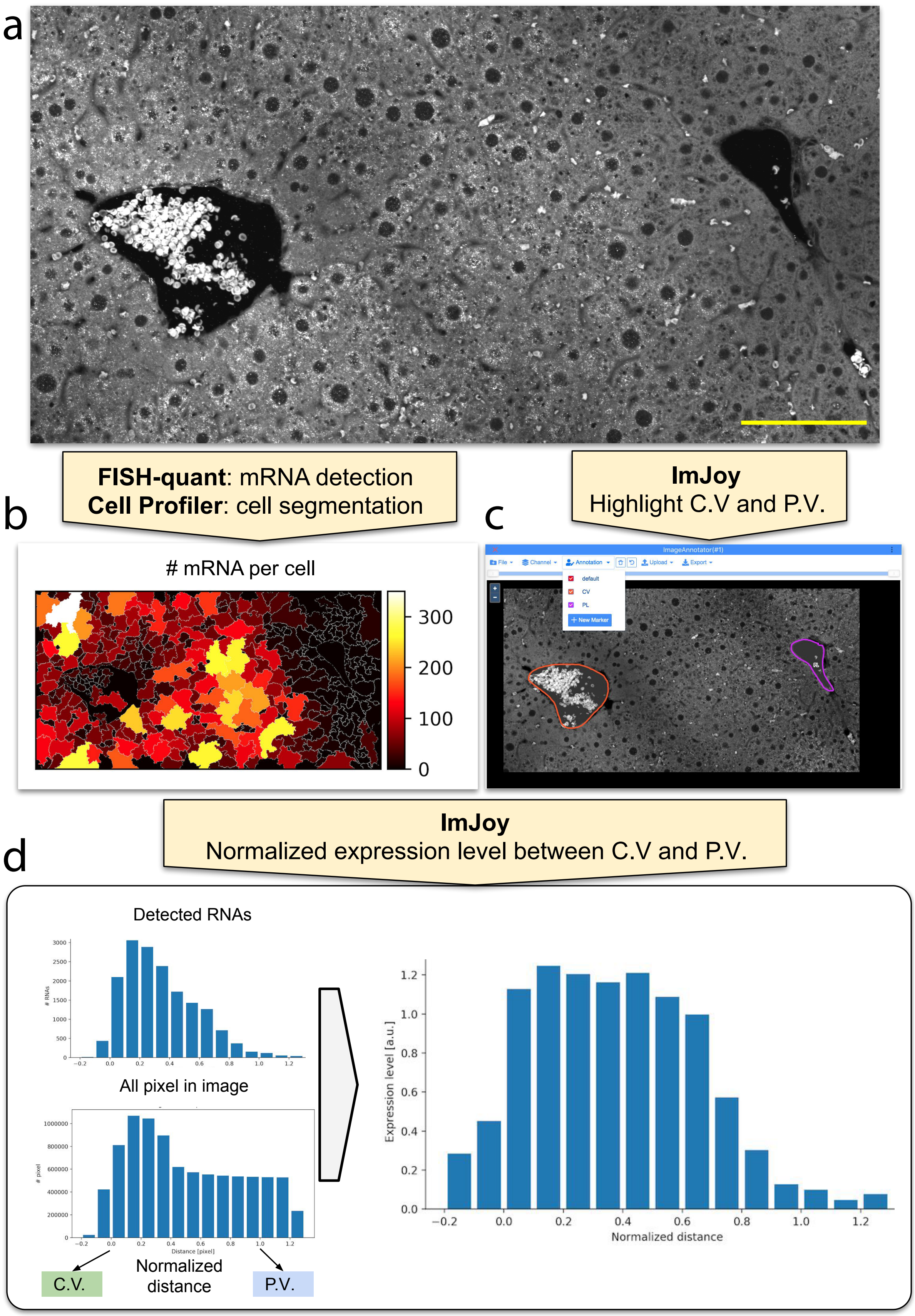

a
FIG.-quant: mRNA detection
FISH-quant: mRNA detection
Cell Profiler: cell segmentation
ImJoy
Highlight C.V and P.V.
b
mRNA per cell
300
200
100
0
c
ImageAnnotator(#1)
d
ImJoy
Normalized expression level between C.V and P.V.
Detected RNAs
All pixel in image
Normalized distance
C.V.
P.V.
Expression level [a.u.]
Normalized distance

Supplementary Fig. 3 | Workflow for smFISH data analysis
Overview summarizing different steps to obtain spatial expression gradients from smFISH images. For more details, please consult the Methods section. Cells were automatically segmented with CellProfiler **(a)** and individual mRNA molecules were detected with FISH-quant **(b)**. **(c)** In each image, the central vein (C.V.) and portal vein (P.V.) were manually annotated as polygons in ImJoy. **(d)** The normalized expression gradients were calculated with an ImJoy plugin as follows: for each RNA the distance to the polygon of the C.V. is calculated (positive values for mRNAs outside the C.V., negative values for mRNAs inside the C.V.). These distances are renormalized with the closest distance between the polygon of the C.V. and the center of mass of the polygon defining the P.V. A normalized distance value of 0 thus corresponds to a position at the C.V., whereas a value of 1 corresponds to a position at the center of mass of the P.V. Renormalized distances are summarized in a histogram with a bin width of 0.1. Finally, this histogram is normalized to consider that not all distances are equally represented in the image, e.g. a proportionally larger region close to the C.V. is present in the image. For this, we calculate the distance of all pixels in the image to the C.V. and summarize these measurements in a histogram as described for the RNA distance. Each bin of the RNA distance histogram is then divided by the corresponding bin of the latter histogram. Scale bar is 100 microns.

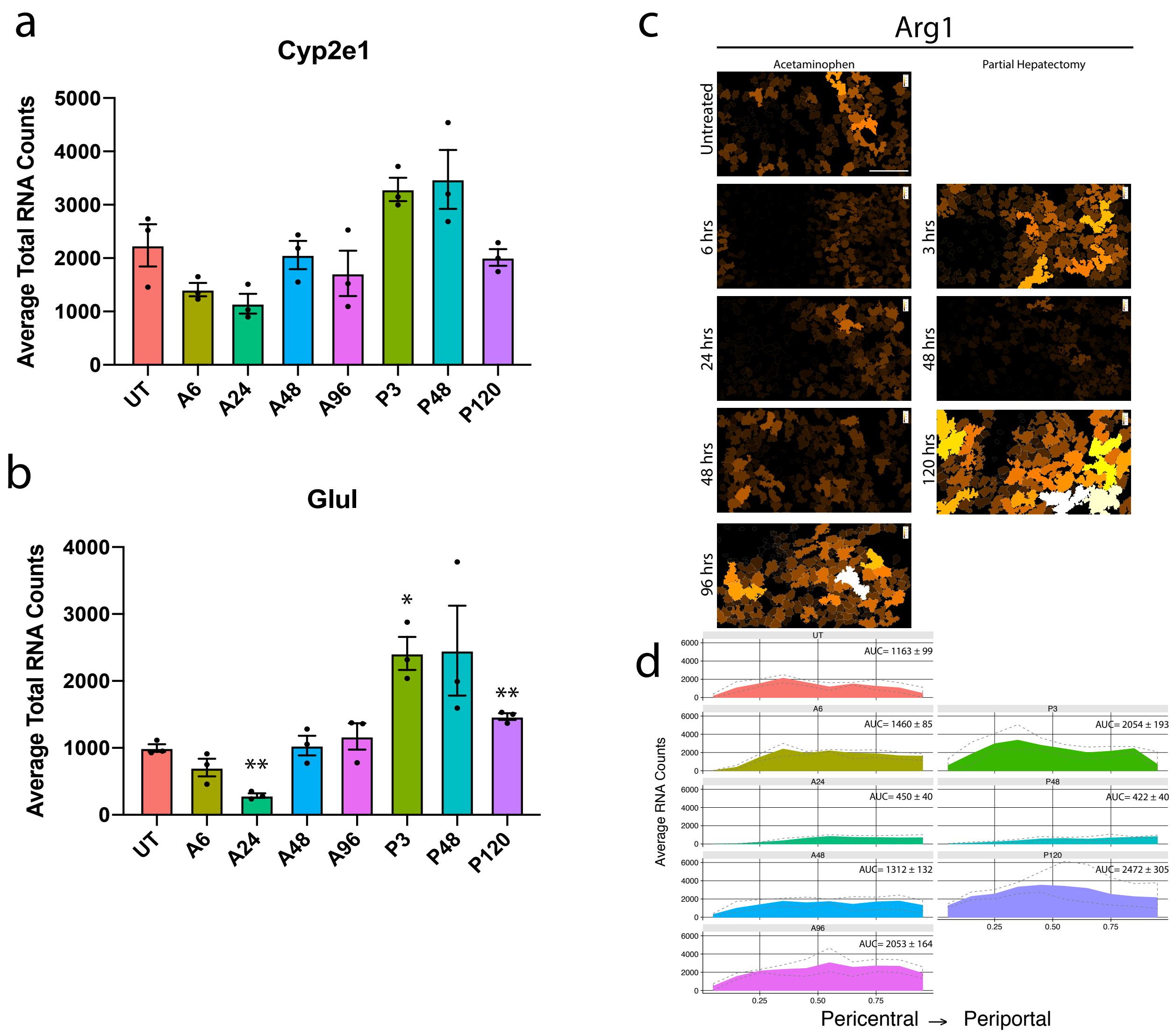

Supplementary Fig. 4 | Average RNA expression of *Cyp2e1* and *Glul* following acute liver injury

Average RNA expression quantified by smFISH of *Cyp2e1* **(a)** and *Glul* **(b)** following injury induced by APAP or PH. smFISH quantification shown as bar plot with individual replicates presented as dots (n=3). Data reflects the analysis of 3 independent liver lobules/time point. Error bars represent s.e.m., $P < 0.05$ (*), < 0.005 (**), < 0.0005 (***), and < 0.0001 (****) calculated using unpaired t tests with Welch's correction (two-tailed). **(c)** Imaging of liver section showing periportal marker *Arg1* for untreated and each APAP-treated or PH-treated time point (left column). Cell outlined and colored by number of *Arg1* transcripts (brown, low; white, high) for each condition. Scale bars is 100 microns. **(d)** Quantification of gene expression intensity across the lobule for *Arg1*. Source data provided as a Source Data file.

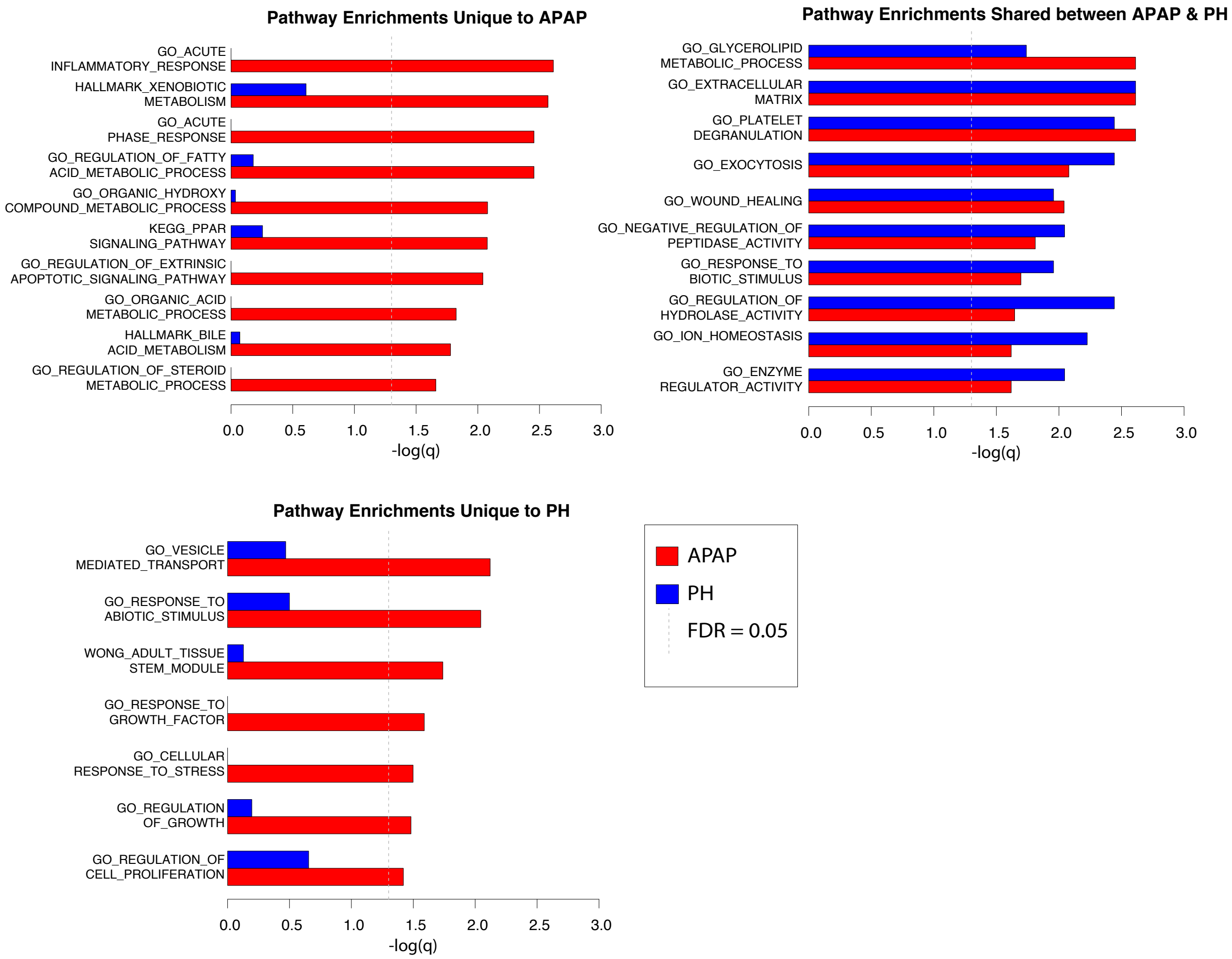

Supplementary Fig. 5 | Pathway Analysis of APAP and PH
Pathways with significant overlaps with differentially expressed genes. Significant pathways unique to APAP response (left), unique to PH response (middle) and significant in both responses (right).

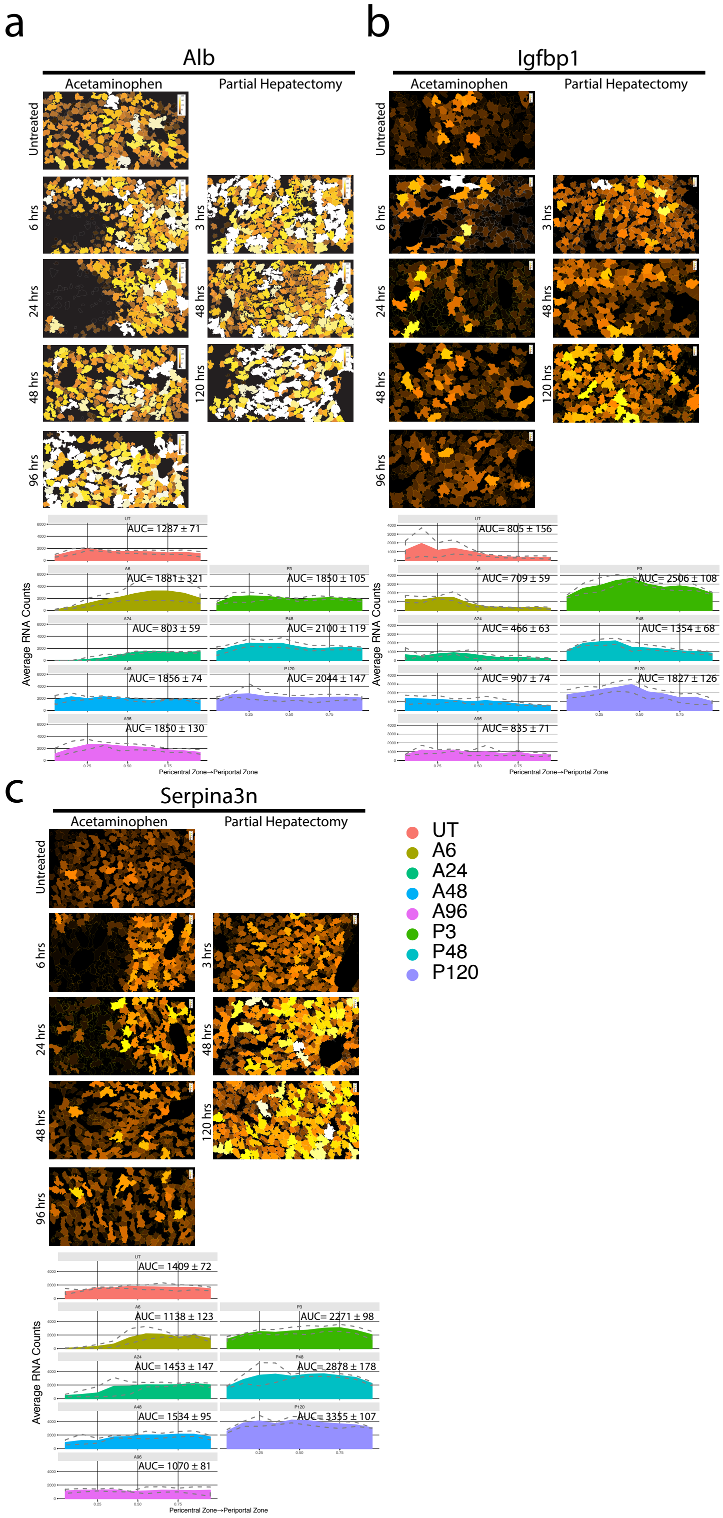
a
Alb
Acetaminophen
Partial Hepatectomy
Untreated
6 hrs
24 hrs
48 hrs
96 hrs
3 hrs
48 hrs
120 hrs
AUC= 1287 ± 71
AUC= 1881 ± 321
AUC= 1850 ± 105
AUC= 803 ± 59
AUC= 2100 ± 119
AUC= 1856 ± 74
AUC= 2044 ± 147
AUC= 1850 ± 130
Average RNA Counts
Pericentral Zone→Periportal Zone
b
Igfbp1
Acetaminophen
Partial Hepatectomy
Untreated
6 hrs
24 hrs
48 hrs
96 hrs
3 hrs
48 hrs
120 hrs
AUC= 805 ± 156
AUC= 709 ± 59
AUC= 2506 ± 108
AUC= 466 ± 63
AUC= 1354 ± 68
AUC= 907 ± 74
AUC= 1827 ± 126
AUC= 835 ± 71
Average RNA Counts
Pericentral Zone→Periportal Zone
c
Serpina3n
Acetaminophen
Partial Hepatectomy
Untreated
6 hrs
24 hrs
48 hrs
96 hrs
3 hrs
48 hrs
120 hrs
AUC= 1409 ± 72
AUC= 1138 ± 123
AUC= 2271 ± 98
AUC= 1453 ± 147
AUC= 2878 ± 178
AUC= 1534 ± 95
AUC= 3355 ± 107
AUC= 1070 ± 81
Average RNA Counts
Pericentral Zone→Periportal Zone
UT
A6
A24
A48
A96
P3
P48
P120

Supplementary Fig. 6 | Hepatocyte gene expression following acute liver injury using smFISH – Secreted Proteins

Imaging of liver section (5 μm) showing spatiotemporal maps of the number of transcripts counted (brown, low; white, high) for hepatic genes corresponding to secreted proteins: **(a)** *Alb*, **(b)** *Igfbp1*, **(c)** *Serpina3n*. Quantification of gene expression intensity (y-axis) across the lobule (x-axis) for each gene can be found below each image set. Total AUC is posted above each plot. Data reflects the analysis of 3 independent liver lobules/time point. Source data provided as a Source Data file.

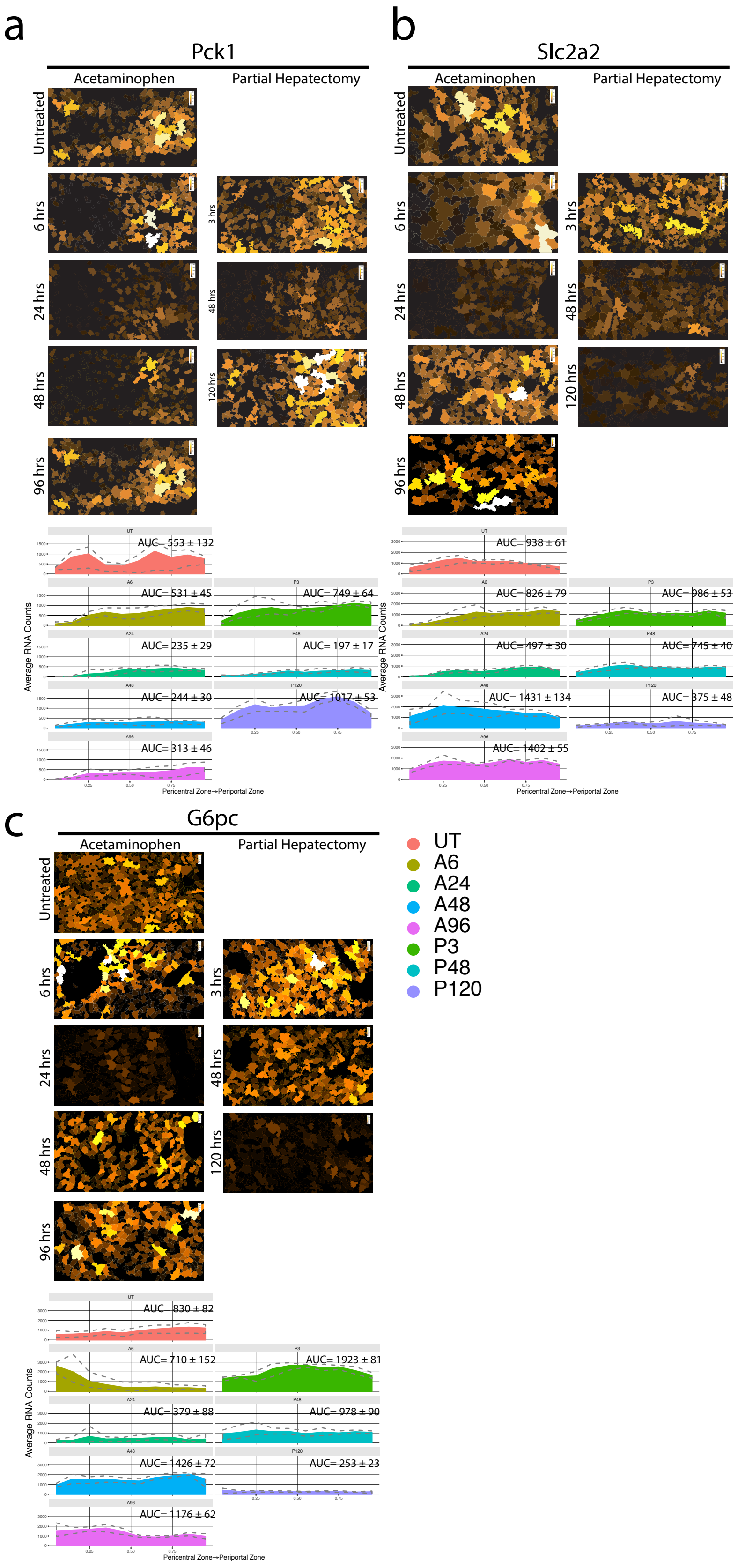
a
Pck1
Acetaminophen
Partial Hepatectomy
Untreated
6 hrs
3 hrs
24 hrs
48 hrs
48 hrs
120 hrs
96 hrs
AUC= 553 ± 132
AUC= 531 ± 45
AUC= 749 ± 64
AUC= 235 ± 29
AUC= 197 ± 17
AUC= 244 ± 30
AUC= 1017 ± 53
AUC= 313 ± 46
Average RNA Counts
Pericentral Zone→Periportal Zone
b
Slc2a2
Acetaminophen
Partial Hepatectomy
Untreated
6 hrs
3 hrs
24 hrs
48 hrs
48 hrs
120 hrs
96 hrs
AUC= 938 ± 61
AUC= 826 ± 79
AUC= 986 ± 53
AUC= 497 ± 30
AUC= 745 ± 40
AUC= 1431 ± 134
AUC= 375 ± 48
AUC= 1402 ± 55
Average RNA Counts
Pericentral Zone→Periportal Zone
c
G6pc
Acetaminophen
Partial Hepatectomy
Untreated
6 hrs
3 hrs
24 hrs
48 hrs
48 hrs
120 hrs
96 hrs
AUC= 830 ± 82
AUC= 710 ± 152
AUC= 1923 ± 81
AUC= 379 ± 88
AUC= 978 ± 90
AUC= 1426 ± 72
AUC= 253 ± 23
AUC= 1176 ± 62
Average RNA Counts
Pericentral Zone→Periportal Zone
UT
A6
A24
A48
A96
P3
P48
P120

Supplementary Fig. 7 | Hepatocyte gene expression following acute liver injury using smFISH – Gluconeogenesis

Imaging of liver section (5 μm) showing spatiotemporal maps of the number of transcripts counted (brown, low; white, high) for hepatic genes corresponding to gluconeogenesis: **(a)** *Pck1*, **(b)** *Slc2a2*, **(c)** *G6pc*. Quantification of gene expression intensity (y-axis) across the lobule (x-axis) for each gene can be found below each image set. Total AUC is posted above each plot. Data reflects the analysis of 3 independent liver lobules/time point. Source data provided as a Source Data file.

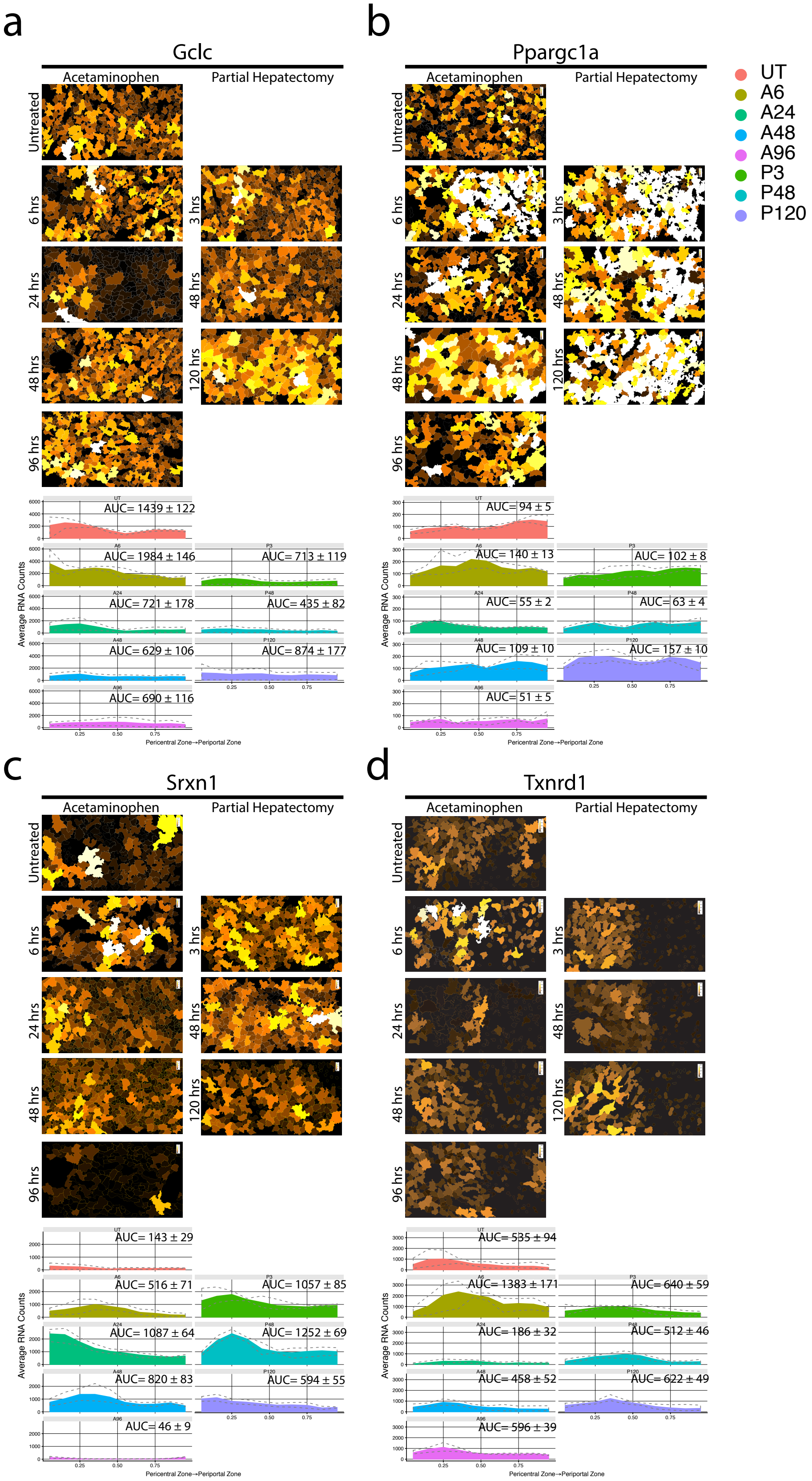

a
Gclc
Acetaminophen
Partial Hepatectomy
Untreated
6 hrs
3 hrs
24 hrs
48 hrs
48 hrs
120 hrs
96 hrs
AUC= 1439 ± 122
AUC= 1984 ± 146
AUC= 713 ± 119
AUC= 721 ± 178
AUC= 435 ± 82
AUC= 629 ± 106
AUC= 874 ± 177
AUC= 690 ± 116
Average RNA Counts
Pericentral Zone→Periportal Zone
b
Ppargc1a
Acetaminophen
Partial Hepatectomy
Untreated
6 hrs
3 hrs
24 hrs
48 hrs
48 hrs
120 hrs
96 hrs
AUC= 94 ± 5
AUC= 140 ± 13
AUC= 102 ± 8
AUC= 55 ± 2
AUC= 63 ± 4
AUC= 109 ± 10
AUC= 157 ± 10
AUC= 51 ± 5
Average RNA Counts
Pericentral Zone→Periportal Zone
UT
A6
A24
A48
A96
P3
P48
P120
c
Srxn1
Acetaminophen
Partial Hepatectomy
Untreated
6 hrs
3 hrs
24 hrs
48 hrs
48 hrs
120 hrs
96 hrs
AUC= 143 ± 29
AUC= 516 ± 71
AUC= 1057 ± 85
AUC= 1087 ± 64
AUC= 1252 ± 69
AUC= 820 ± 83
AUC= 594 ± 55
AUC= 46 ± 9
Average RNA Counts
Pericentral Zone→Periportal Zone
d
Txnrd1
Acetaminophen
Partial Hepatectomy
Untreated
6 hrs
3 hrs
24 hrs
48 hrs
48 hrs
120 hrs
96 hrs
AUC= 535 ± 94
AUC= 1383 ± 171
AUC= 640 ± 59
AUC= 186 ± 32
AUC= 512 ± 46
AUC= 458 ± 52
AUC= 622 ± 49
AUC= 596 ± 39
Average RNA Counts
Pericentral Zone→Periportal Zone

Supplementary Fig. 8 | Hepatocyte gene expression following acute liver injury using smFISH – Metabolism

Imaging of liver section (5 μm) showing spatiotemporal maps of the number of transcripts counted (brown, low; white, high) for hepatic genes corresponding to metabolism: **(a)** *Gclc*, **(b)** *Ppargc1a*, **(c)** *Srxn1*, **(d)** *Txnrd1*. Quantification of gene expression intensity (y-axis) across the lobule (x-axis) for each gene can be found below each image set. Total AUC is posted above each plot. Data reflects the analysis of 3 independent liver lobules/time point. Source data provided as a Source Data file.

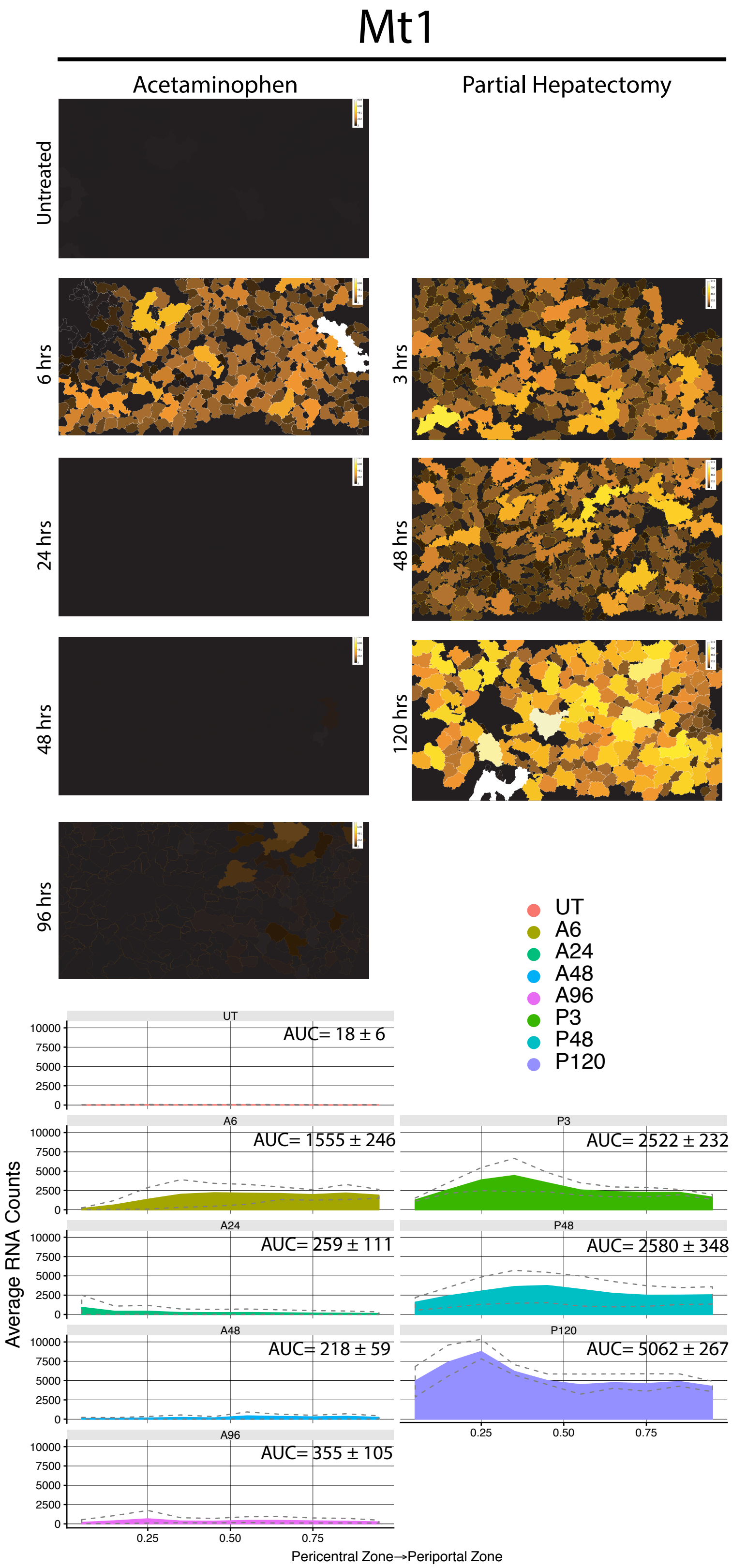
Mt1
Acetaminophen
Partial Hepatectomy
Untreated
6 hrs
3 hrs
24 hrs
48 hrs
48 hrs
120 hrs
96 hrs
UT
A6
A24
A48
A96
P3
P48
P120
AUC= 18 ± 6
AUC= 1555 ± 246
AUC= 2522 ± 232
AUC= 259 ± 111
AUC= 2580 ± 348
AUC= 218 ± 59
AUC= 5062 ± 267
AUC= 355 ± 105
Average RNA Counts
Pericentral Zone→Periportal Zone

Supplementary Fig. 9 | Hepatocyte gene expression following acute liver injury using smFISH – Ion Homeostasis

Imaging of liver section (5 μm) showing spatiotemporal maps of the number of transcripts counted (brown, low; white, high) for hepatic genes corresponding to ion homeostasis (*Mt1*). Quantification of gene expression intensity (y-axis) across the lobule (x-axis) can be found below each image set. Total AUC is posted above each plot. Data reflects the analysis of 3 independent liver lobules/time point. Source data provided as a Source Data file.

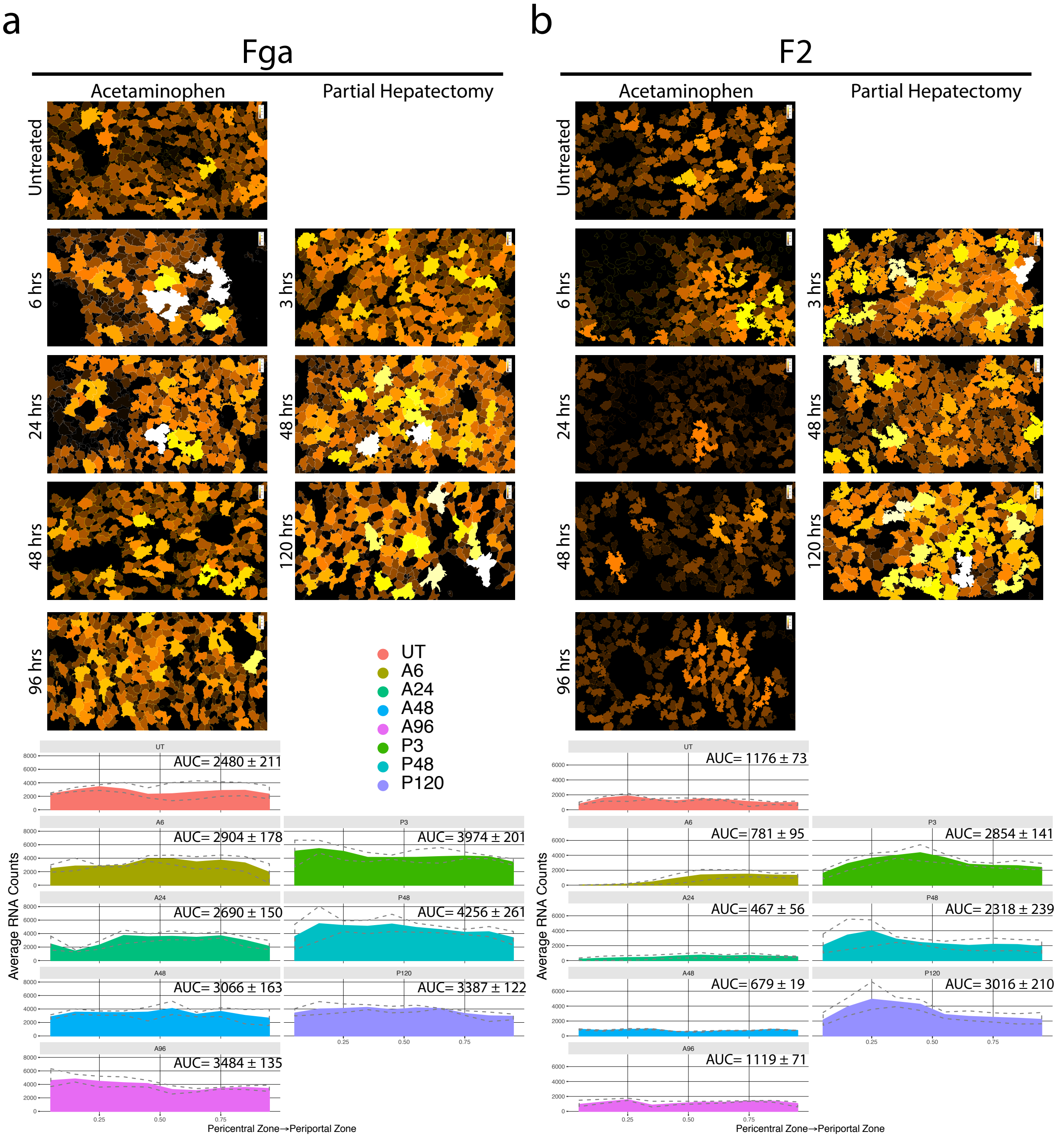

a
Fga
Acetaminophen
Partial Hepatectomy
Untreated
6 hrs
24 hrs
48 hrs
96 hrs
3 hrs
48 hrs
120 hrs
UT
A6
A24
A48
A96
P3
P48
P120
AUC= 2480 ± 211
AUC= 2904 ± 178
AUC= 3974 ± 201
AUC= 2690 ± 150
AUC= 4256 ± 261
AUC= 3066 ± 163
AUC= 3387 ± 122
AUC= 3484 ± 135
Average RNA Counts
Pericentral Zone→Periportal Zone
b
F2
Acetaminophen
Partial Hepatectomy
Untreated
6 hrs
24 hrs
48 hrs
96 hrs
3 hrs
48 hrs
120 hrs
AUC= 1176 ± 73
AUC= 781 ± 95
AUC= 2854 ± 141
AUC= 467 ± 56
AUC= 2318 ± 239
AUC= 679 ± 19
AUC= 3016 ± 210
AUC= 1119 ± 71
Average RNA Counts
Pericentral Zone→Periportal Zone

Supplementary Fig. 10 | Hepatocyte gene expression following acute liver injury using smFISH – Blood Clotting

Imaging of liver section (5 μm) showing spatiotemporal maps of the number of transcripts counted (brown, low; white, high) for hepatic genes corresponding to blood clotting: **(a)** *Fga* and **(b)** *F2*. Quantification of gene expression intensity (y-axis) across the lobule (x-axis) for each gene can be found below each image set. Total AUC is posted above each plot. Data reflects the analysis of 3 independent liver lobules/time point. Source data provided as a Source Data file.

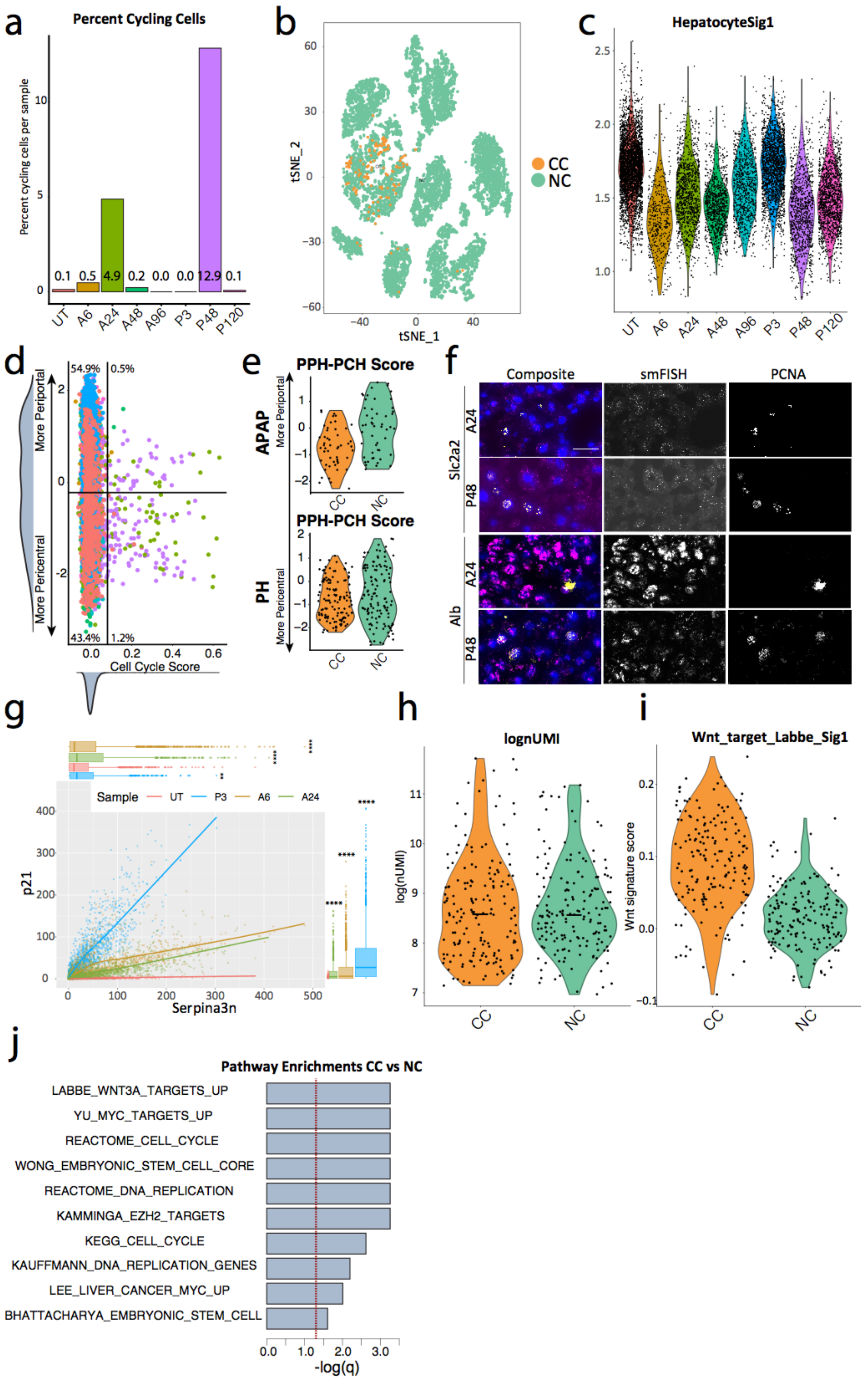
a
Percent Cycling Cells
Percent cycling cells per sample
0.1 0.5 4.9 0.2 0.0 0.0 12.9 0.1
UT A6 A24 A48 A96 P3 P48 P120
b
tSNE_1
tSNE_2
CC
NC
c
HepatocyteSig1
UT A6 A24 A48 A96 P3 P48 P120
d
More Periportal
More Pericentral
54.9% 0.5%
43.4% 1.2%
Cell Cycle Score
e
PPH-PCH Score
APAP
PH
More Periportal
More Pericentral
CC NC
f
Composite
smFISH
PCNA
Slc2a2
Alb
A24
P48
g
Sample UT P3 A6 A24
p21
Serpina3n
h
lognUMI
log(nUMI)
CC NC
i
Wnt_target_Labbe_Sig1
Wnt signature score
CC NC
j
Pathway Enrichments CC vs NC
LABBE_WNT3A_TARGETS_UP
YU_MYC_TARGETS_UP
REACTOME_CELL_CYCLE
WONG_EMBRYONIC_STEM_CELL_CORE
REACTOME_DNA_REPLICATION
KAMMINGA_EZH2_TARGETS
KEGG_CELL_CYCLE
KAUFFMANN_DNA_REPLICATION_GENES
LEE_LIVER_CANCER_MYC_UP
BHATTACHARYA_EMBRYONIC_STEM_CELL
-log(q)

Supplementary Fig. 11 | Cycling Cells Figure

(a) Barplot of percentage of hepatocytes from each treatment condition which were classified as cycling cells. **(b)** t-SNE of all hepatocytes classified as cycling cells (CC, orange) or non-cycling cells (NC, aqua). **(c)** Violin plot of hepatocyte signature score (module score calculated over a list of hepatocyte genes) grouped by treatment condition. **(d)** Scatter plot of Periportal-pericentral Score (positive more periportal, negative more pericentral) versus Cell Cycle Score. Horizonal line represents average Periportal-pericentral Score calculated over all untreated cells. Vertical line represents two standard deviations above the average cell cycle score. **(e)** Periportal-pericentral Score for for cycling (CC) and non-cycling (NC) cells in A24 (top; p = 2.7e-04; Cohen's d effect size = -0.85) and PH48 (lower; p = 0.25; Cohen's d effect size = -0.17). **(f)** Co-staining of PCNA (IF) and smFISH for both *Alb* and *Slc2a2* at A24 and P48. Scale bar is 30 microns. Data reflects the analysis of 3 independent lobules/group. **(g)** Quantification of RNA counts of p21 versus *Serpina3n*. Box plots represent data for both axes. smFISH co-staining tested using one-way ANOVA ($P < 0.0001$) and Dunnett's Multiple Comparisons Test (****, $P < 0.0001$). **(h-i)** violin plot lognUMI **(h)** and Wnt target genes **(i)** for cycling (CC) and non-cycling (NC) cells for A24 and PH48. **(j)** Pathway analysis of differentially expressed genes between CC and NC in APAP 24hr and PH48 hr. Source data provided as a Source Data file.

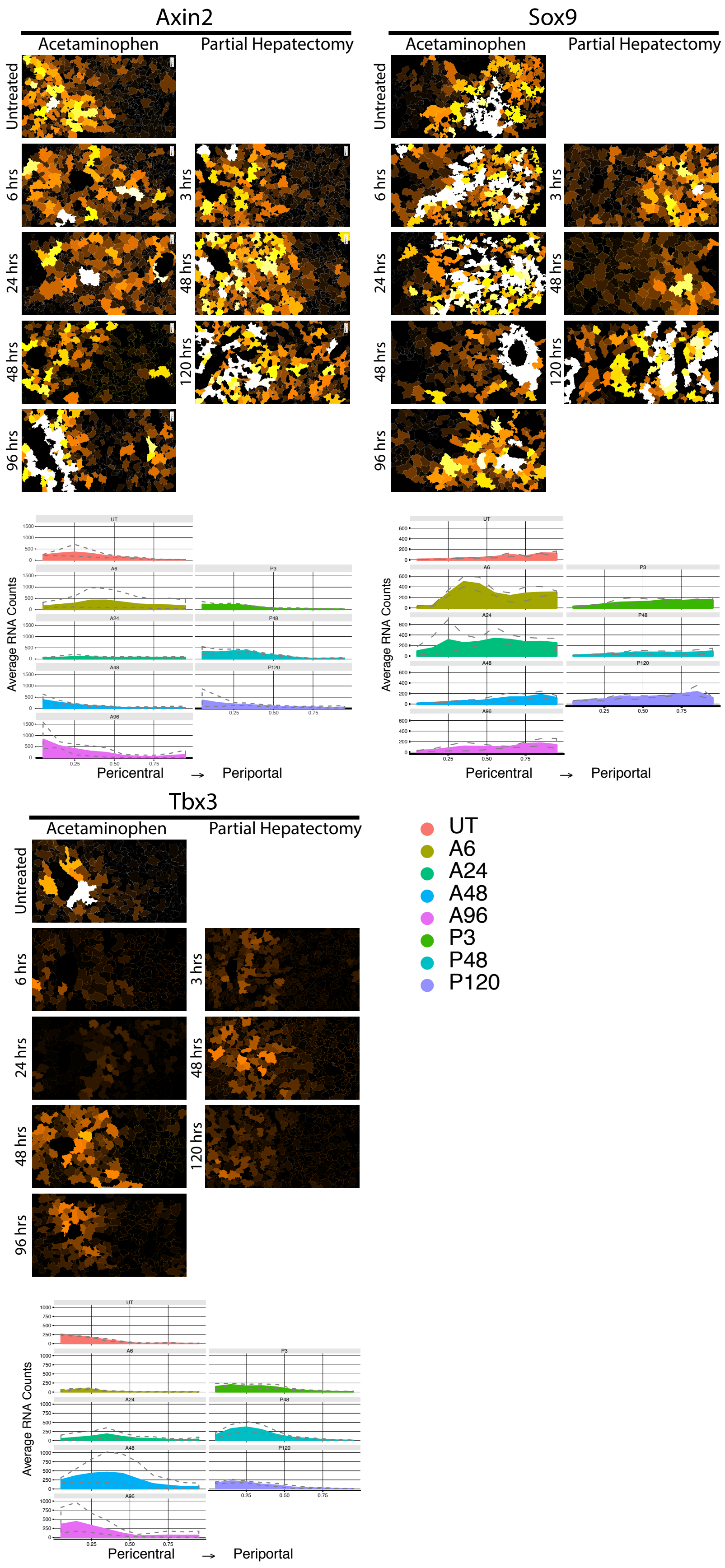
Axin2
Acetaminophen
Partial Hepatectomy
Untreated
6 hrs
3 hrs
24 hrs
48 hrs
48 hrs
120 hrs
96 hrs
Average RNA Counts
Pericentral
→
Periportal
Sox9
Tbx3
UT
A6
A24
A48
A96
P3
P48
P120

Supplementary Fig. 12 | Expression of putative hepatic stem cell markers following acute liver injury using smFISH

Imaging of liver section (5 μm) showing spatiotemporal maps of the number of transcripts counted (brown, low; white, high) for putative hepatic stem cell markers *Axin2*, *Sox9*, and *Tbx3*. Quantification of gene expression intensity (y-axis) across the lobule (x-axis) for each gene can be found below each image set. Total AUC is posted above each plot. Data reflects the analysis of 3 independent lobules/group. Source data provided as a Source Data file.

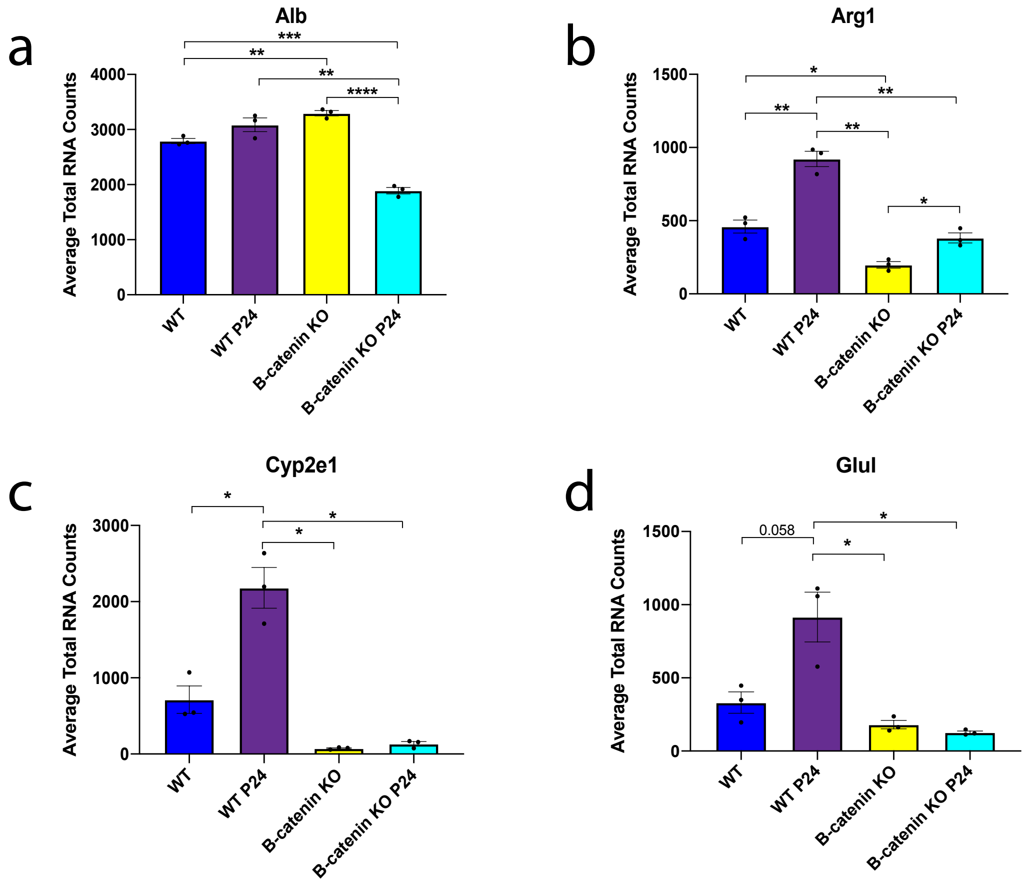

Supplementary Fig. 13 | Expression of hepatic genes in β-catenin KO mice following PH

Average RNA expression of hepatic genes *Alb* **(a)**, *Arg1* **(b)**, *Cyp2e1* **(c)**, and *Glul* **(d)** in WT and β-catenin KO mice in control and 24 hrs after PH. Functional compensation of *Alb* and *Glul* appears to be dependent on β-catenin, whereas compensation of *Arg1* and *Cyp2e1* is independent. smFISH quantification shown as bar plot with individual replicates presented as dots (n=3). Error bars are s.e.m., $P < 0.05$ (*), < 0.005 (**), < 0.0005 (***), and < 0.0001 (****) calculated using unpaired t tests with Welch's correction (two-tailed). Source data provided as a Source Data file.

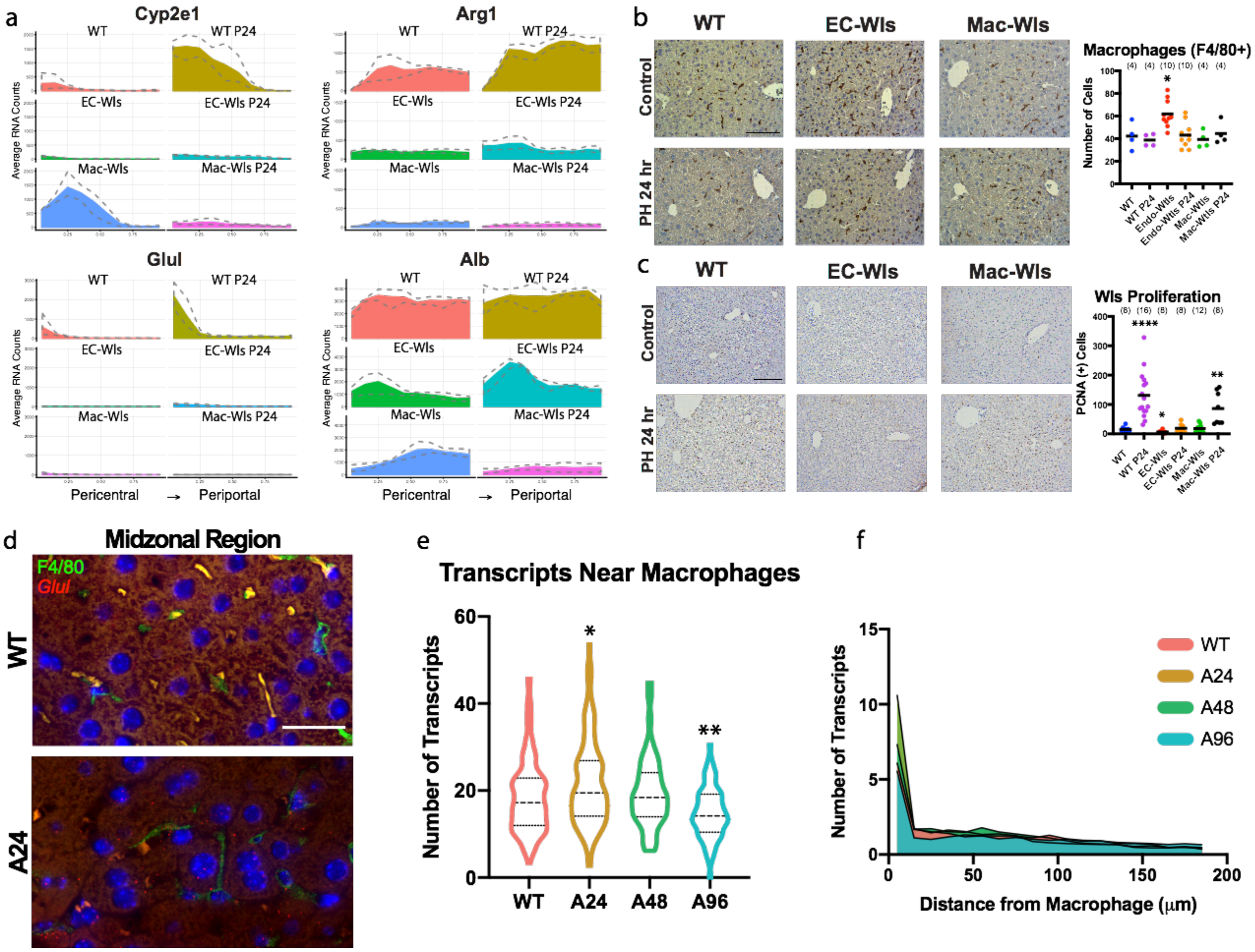

Supplementary Fig. 14 | Quantification of macrophages in Wntless KO mice

(a) Quantification of gene expression intensity across the lobule for *Cyp2e1*, *Arg1*, *Glul*, and *Alb* for WT, EC-*Wls* KO, and Mac-*Wls* KO mice. Represented is control versus 24 hrs following PH. **(b)** IHC staining for macrophages (F4/80+) in WT, EC-*Wls* KO, and Mac-*Wls* KO mice in control and 24 hrs after PH. Quantification of macrophages (F4/80+) from 40x fields represented as dot plot. Scale bar is 30 microns. The number of replicates (n) presented in figure panel for each group. $P < 0.05$ (*), < 0.005 (**), < 0.0005 (***), and < 0.0001 (****) calculated using unpaired t tests with Welch's correction (two-tailed). **(c)** IHC staining for proliferating cells (PCNA) in WT, EC-*Wls* KO, and Mac-*Wls* KO mice in control and 24 hrs after PH. Quantification of proliferating cells (PCNA) from 40x fields represented as dot plot. Scale bar is 100 microns. The number replicates (n) presented in figure panel for each group. $P < 0.05$ (*), < 0.005 (**), < 0.0005 (***), and < 0.0001 (****) calculated using unpaired t tests with Welch's correction (two-tailed). **(d)** **I**mmunofluorescence (F480+ macrophages, green) coupled with smFISH for *Glul* (red puncta) for both WT and A24. Scale bar is 30 microns. Data reflects the analysis of 3 independent lobules/group. **(e)** Quantification of *Glul* transcript within ~30 microns of each detected F4/80+ macrophage. Tested using the Kolmogorov-Smirnov test (two-tailed), $P < 0.05$ (*), < 0.01 (**). **(f)** Histogram showing the average number of transcripts detected versus distance (μm) from each detected F4/80+ macrophage. Source data provided as a Source Data file.
