# Supplementary material for: Functional compensation precedes recovery of tissue mass following acute liver injury
Source: Nat Commun. 2020 Nov 19;11:5785. doi: 10.1038/s41467-020-19558-3 (PMC7677389; doi:10.1038/s41467-020-19558-3)
Supplement: Supplementary file 3 — Description of Additional Supplementary Files [file 41467_2020_19558_MOESM3_ESM.pdf]

Description of Additional Supplementary Files

File Name: Supplementary Data 1
Description: Sequencing metrics table containing Average reads, Average genes, Average UMIs, and total number of cells passing quality and hepatocyte identitiy filtering for each sample in the dataset.

File Name: Supplementary Data 2
Description: Differentially expressed genes (DEG) between the three untreated (UT) samples. Calculated using the FindAllMarkers function in the R package Seurat using the Wilcox test (two-sided).

File Name: Supplementary Data 3
Description: DEG for each treatment condition vs UT. Positive log fold-change (avg_logFC) values indicate a gene up-regulated in the treatment conditions, negative values indicate genes up-regulated in the UT condition. Calculated using the FindMarkers function in the R package Seurat using the Wilcox test (two-sided).

File Name: Supplementary Data 4
Description: Composite DEG for partial hepatectomy (PH) and acetaminophen (APAP) treatment. DEG for each individual treatment condition (Supplementary Table 3) were pooled across all time points within PH conditions and all time points within APAP conditions to generate a composite list of all genes which are differentially expressed at any time point relative to untreated for each injury type. *P* values were calculated using the Wilcox test (two-sided).

File Name: Supplementary Data 5
Description: Shared composite DEG. Table of genes with concordant (up or down) regulation relative to untreated in both APAP and PH. NA indicates that a particular gene was not significantly differentially expressed. *P* values were calculated using the Wilcox test (two-sided).

File Name: Supplementary Data 6
Description: Gene enrichment analysis (GEA) was performed using the R package piano and 3 reference geneset collections from the Broad's MSigDB. Results for composite APAP vs UT, composite PH vs UT, and cycling cells vs noncycling cells (CC vs NC) are included. *P* values were calculated using the Wilcox test (two-sided).

File Name: Supplementary Data 7
Description: DEG for each treatment between cycling cells vs noncycling cells (CC vs NC). Positive log fold-change (avg_logFC) values indicate genes up-regulated in the CCs, negative values indicate genes up-regulated in the NCs. Calculated using the FindMarkers function in the R package Seurat using the Wilcox test (two-sided).

File Name: Supplementary Data 8
Description: Gene signatures used to define pericentral hepatocytes (PCH) and periporatal hepatocytes (PPH).
